# SEE SOMETHING, SAY SOMETHING: A QUALITATIVE STUDY OF NEIGHBORHOOD PERCEPTIONS AND BRAIN HEALTH

**DOI:** 10.1093/geroni/igae098.4257

**Published:** 2024-12-31

**Authors:** Sierra Heuer, Lilah Besser, Briana Dominguez, Stephanie Huynh, Bao Ngoc Le, Boi-san Nguyen, Jason Pham, Oanh Meyer

**Affiliations:** University of California, Davis, Sacramento, California, United States; University of Miami, Boca Raton, Florida, United States; University of California, Davis, Sacramento, California, United States; University of California, Davis, Sacramento, California, United States; University of California, Davis, Sacramento, California, United States; University of California, Davis, Sacramento, California, United States; University of California, Davis, Sacramento, California, United States; University of California, Davis, Sacramento, California, United States

## Abstract

Studies have shown the importance of neighborhood factors, such as residential segregation and green spaces, to older adult brain health. In this qualitative study, we examine older adults’ perceptions of their neighborhood, with a particular focus on segregation. We recruited participants from the University of California Davis Alzheimer’s Disease Research Center and the local Northern California community to participate in semi-structured interviews over Zoom or telephone. They answered questions such as, “What kinds of activities do you do in your neighborhood?” and “Are neighbors segregated or separated based on age, race/ethnicity, SES, or language?” On average, interviews lasted 22.5 minutes, and were audiotaped, transcribed verbatim, and analyzed for recurring themes. Among the 32 participants, 62.5% were female, 59.4% non-Hispanic White, and 40.6% Black/African American. Mean age was 79.0 years (SD=8.3). The most prominent themes were (1) Accessibility to public stores and similar third spaces (e.g., churches, libraries, coffee shops); (2) Accessibility to green space, including parks, trees, etc.; (3) Staying active in the community, both physically and socially; (4) Neighborhood awareness/cohesion; and (5) Stereotypes, discrimination, and segregation, a sample quote being, “I’m happy with the diversity of the neighborhood…a lot of people would say it’s not very diverse, it’s…segregated, mostly economically,” (Male, 73 years-old). These findings highlight the social and built environment factors that are salient for older adults and may potentially affect brain health. Future research will examine how these themes might differ depending on older adults’ cognition and with objective measures of the neighborhood.

